# Heterozygous Mutation of Opa1 in *Drosophila* Shortens Lifespan Mediated through Increased Reactive Oxygen Species Production

**DOI:** 10.1371/journal.pone.0004492

**Published:** 2009-02-16

**Authors:** Sha Tang, Phung Khanh Le, Stephanie Tse, Douglas C. Wallace, Taosheng Huang

**Affiliations:** 1 Division of Human Genetics, Department of Pediatrics, University of California Irvine, Irvine, California, United States of America; 2 Center for Molecular and Mitochondrial Medicine and Genetics, University of California Irvine, Irvine, California, United States of America; 3 Department of Biological Chemistry, University of California Irvine, Irvine, California, United States of America; 4 Department of Developmental and Cell Biology, University of California Irvine, Irvine, California, United States of America; 5 Department of Pathology, University of California Irvine, Irvine, California, United States of America; University Medical Center Groningen, Netherlands

## Abstract

Optic atrophy 1 (OPA1) is a dynamin-like GTPase located in the inner mitochondrial membrane and mutations in *OPA1* are associated with autosomal dominant optic atrophy (DOA). OPA1 plays important roles in mitochondrial fusion, cristae remodeling and apoptosis. Our previous study showed that *dOpa*1 mutation caused elevated reactive oxygen species (ROS) production and resulted in damage and death of the cone and pigment cells in *Drosophila* eyes. Since ROS-induced oxidative damage to the cells is one of the primary causes of aging, in this study, we examined the effects of heterozygous *dOpa*1 mutation on the lifespan. We found that heterozygous *dOpa*1 mutation caused shortened lifespan, increased susceptibility to oxidative stress and elevated production of ROS in the whole *Drosophila*. Antioxidant treatment partially restored lifespan in the male *dOpa1* mutants, but had no effects in the females. Heterozygous *dOpa*1 mutation caused an impairment of respiratory chain complex activities, especially complexes II and III, and reversible decreased aconitase activity. Heterozygous *dOpa*1 mutation is also associated with irregular and dysmorphic mitochondria in the muscle. Our results, for the first time, demonstrate the important role of OPA1 in aging and lifespan, which is most likely mediated through augmented ROS production.

## Introduction

OPA1 is a ubiquitously expressed large dynamin-related GTPase, encoded by the nuclear genome and targeted to the inner mitochondrial membrane [1]–[3]. The predicted structure of OPA1 reveals a mitochondrial targeting signal, a transmembrane domain, a presenilin-associated rhomboid-like protease (PARL) recognition site, and a dynamin/GTPase domain. The mitochondrial inner membrane PARL is required for the correct proteolytical processing of OPA1 and subsequent assembly of OPA1 oligomers [4]. OPA1 promotes mitochondrial fusion and is essential for maintaining normal mitochondrial morphology. In addition, OPA1 regulates cytochrome c mediated apoptosis by modulating mitochondrial cristae structures [5], [6].

In human, mutations of OPA1 cause dominant optic atrophy (DOA), the most common form of autosomal inherited optic neuropathy. DOA is characterized by degeneration of retinal ganglion cells [7] and progressive loss of vision [8]. Optical atrophy can be accompanied by deafness and/or neurological manifestations [9]–[12]. The most prevalent form of DOA is caused by mutations in the OPA1 protein [13]. Although a substantial progress has been made to unravel the molecular function of OPA1, the pathogenesis of DOA remains poorly understood. Recently, we have generated a *Drosophila* knockout model for optic atrophy [14]. Heterozygous mutation of *dOpa1* by a P-element or transposon insertions caused no discernable eye phenotype under a light microscope, whereas the homozygous mutation resulted in embryonic lethality. In the eye-specific somatic clones, the somatic homozygous mutation of *dOpa1* in the eyes caused rough (mispatterning) and glossy (decreased lens and pigment deposition) eye phenotypes in adult *Drosophila*. In *Drosophila* eyes, *dOpa*1 mutation resulted in elevated ROS generation and mitochondrial fragmentation associated with damage and death of the cone and pigment cells. Treatment with antioxidants or superoxide dismutase (SOD1), as well as over-expression of human SOD1 did reverse the glossy phenotype, further indicating the important role of ROS in etiology of optic atrophy.

ROS remain as one of the most widely accepted cause of aging. Mitochondrial dysfunction was shown to affect longevity [4], [15]. Aging in biological systems is also accompanied by mitochondrial morphological changes [15]. Reducing mitochondrial fission in two fungi models resulted in decreased mitochondrial fragmentation and extended lifespan by increased resistance to induction of apoptosis [16]. Since OPA1 is ubiquitously expressed and *OPA1* mutation is associated with altered mitochondrial dynamics and elevated ROS production in the *Drosophila* retinal cells, OPA1 insufficiency may also accelerate aging and affect lifespan. In this study, we further exploited our *Drosophila* model to investigate the effects of *dOpa*1 mutation on ROS production in *Drosophila*, mitochondrial complex activities and lifespan. We found that heterozygous mutation of *dOpa*1 results in a shortened lifespan, an increased production of ROS, sensitivity to oxidative stress, defects in complex activity of respiratory chain and aberrant mitochondrial morphology. Our studies suggest that heterozygous mutation of *dOpa1* causes shortened lifespan mediated through increased ROS production.

## Results

### Heterozygous mutation of *dOpa1* results in decreased lifespan in *Drosophila*

**Figure 1:**
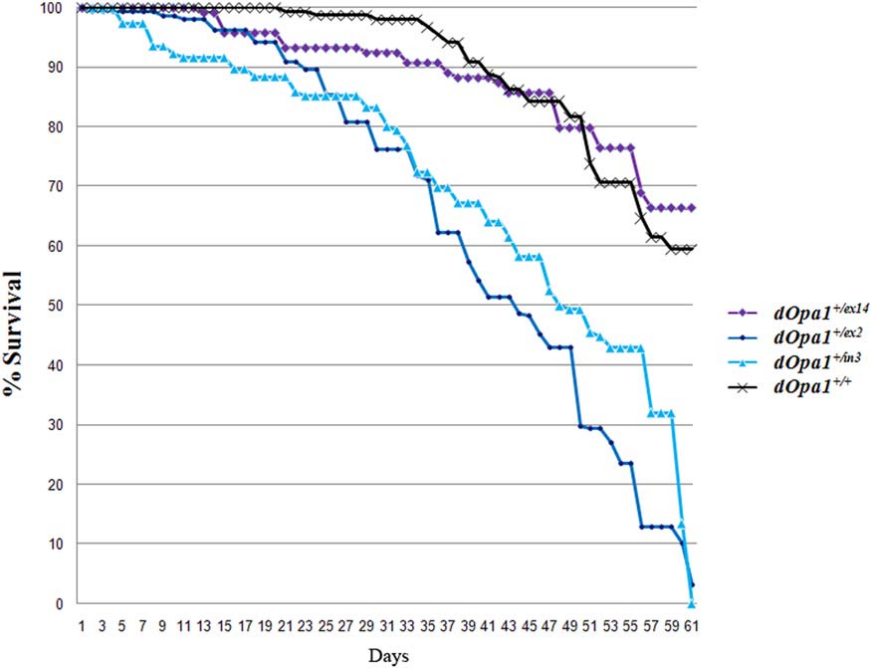
Heterozygous *dOpa1* mutations shorten lifespan in *Drosophila*. The survival curves for *Drosophila* with P Element insertions in exon2 (*dOpa1^+/ex2^*), intron 3 (*dOpa1^+/in3^*), and exon 14 (*dOpa1^+/ex14^*) of *dOpa1*, as well as wild-type (*dOpa1^+/+^*) show that heterozygous mutations in exon 2 and intron 3 of *dOpa1* significantly reduced both median and maximum lifespan. All *dOpa1^+/ex2^* and *dOpa1^+/in3^ Drosophila* had died by Day 61.

To test the hypothesis that heterozygous mutation of *dOpa1* affects the lifespan in *Drosophila*, we performed a longevity assay on a large cohort (n = 300/genotype) of *dOpa1^+/−^* (*dOpa1^+/in3^*, *dOpa1^+/ex2^*) and *dOpa1^+/+^* (*dOpa1^+/+^* and *dOpa1^+/ex14^*) control *Drosophila*. We previously showed that the P-element insertion in exon 2 (*dOpa1* ^+/ex2^) and transposon insertion in intron 3 (*dOpa1* ^+/in3^) disrupted *dOpa1* expression while insertion in non-coding exon 14 had no effect on the dOpa1 protein level, therefore, *dOpa1* ^+/ex14^ also served as a control. *Drosophila* were maintained at 40–50 *Drosophila*/vial, transferred to fresh food and counted every 3 days. As shown in Figure 1, the *dOpa1^+/−^* mutant strain had a significant reduction in both average and maximum lifespan. Since there is no phenotypical difference between *dOpa1^+/in3^* and *dOpa1^+/ex2^*, *dOpa1^+/in3^* is referred as *dOpa1^+/−^* in all subsequent experiments. Wild-type *Drosophila* with the same background are used as the control and referred as *dOpa1^+/+^*.

### Heterozygous mutation of *dOpa1* results in decreased resistance to oxidative stress and increased reactive oxygen species (ROS)

**Figure 2:**
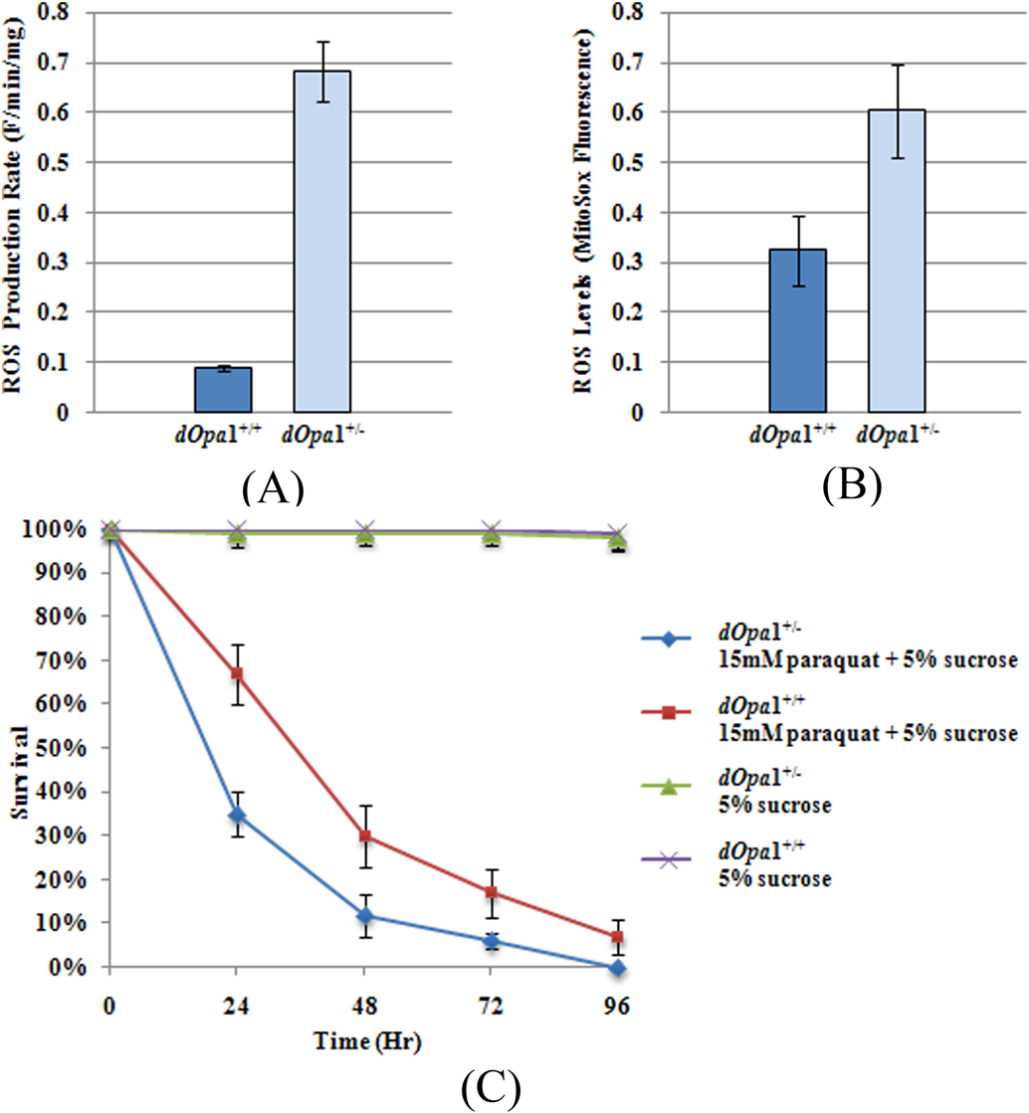
Heterozygous *dOpa1* mutation results in increased Reactive Oxygen Species (ROS) production and decreased resistance to oxidative stress. ROS production rate (A) and ROS levels (B) in *dOpa1^+/−^* and *dOpa1^+/+^ Drosophila* were measured by MitoSOX fluorescence. Survival curves of *dOpa1^+/−^* and *dOpa1^+/+^ Drosophila* exposed to 15 mM paraquat (C) indicated that *dOpa1* deficiency was associated with increased sensitivity to paraquat treatment.

ROS damage biological macromolecules and have been shown to be a major cause of aging. In a previous study, we found that somatically generated homozygous *dOpa*1 mutation large clones displayed higher ROS levels in *dOpa^+/−^* cells than in *dOpa1^−/−^* cells in the eyes [14]. In this experiment, we used MitoSOX to measured the ROS levels of 7 day old *dOpa1^+/−^* and *dOpa1^+/+^* whole flies, and observed that both ROS levels and generation rates were significantly elevated in *dOpa1^+/−^ Drosophila* (Figure 2A, B).

One important genetic determinant for the lifespan of an organism is its sensitivity to oxidative stress [17]. Paraquat can generate more superoxide anion that can lead to synthesis of more ROS [18]. Susceptibility to paraquat can indicate the tolerance of the organism to oxidative stress. To test the overall fitness of *dOpa1* ^+/−^ compared to *dOpa*1^+/+^ *Drosophila*, we exposed adult flies to paraquat for a prolonged period of time and monitored their survival. Compared to control animals, *dOpa1* ^+/−^ *Drosophila* exhibited a significantly increased sensitivity to oxidative stress (Figure 2C).

### Heterozygous mutation of *dOpa1* leads to a respiratory defect in Complex II and III of the electron transport chain (ETC)

**Figure 3:**
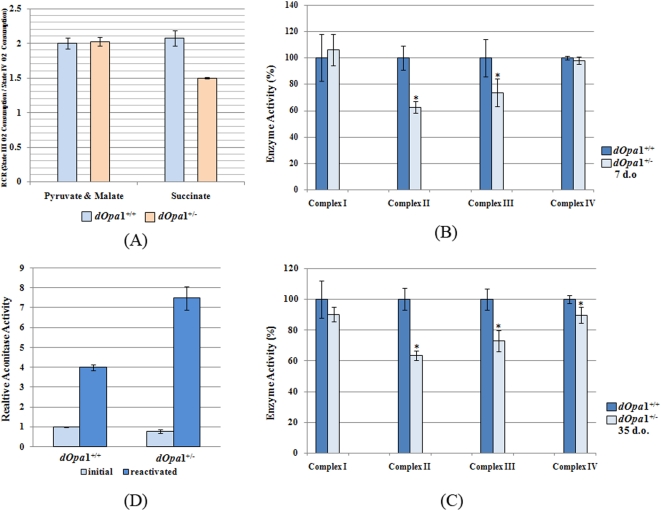
Heterozygous *dOpa1* mutation impairs mitochondrial bioenergetics. (A) *dOpa1^+/−^ Drosophila* displayed compromised oxygen consumption driven by complex II substrate succinate. Mitochondrial respiratory chain complexes II and III activities were also significantly attenuated in both 7 d.o. (B) and 35 d.o. (C) *dOpa1^+/−^ Drosophila*. In the 35-day old *dOpa1^+/−^* flies, mitochondrial aconitase activities were reduced by 22% relative to age-matched controls (D).

In order to further investigate the mechanism by which *dOpa1* mutation elevates ROS, we performed a detailed comparison of respiration of the mitochondria of *dOpa1^+/−^* and *dOpa1^+/+^* adult *Drosophila* (7 d.o.), as inhibition of the mitochondrial ETC can increase ROS. In oxygen consumption assays, metabolism of NADH-linked complex I substrates pyruvate and malate was unaffected by *dOpa1* ^+/−^ mutation while succinate-driven respiration via complex II was significantly decreased (P<0.05) in *dOpa1^+/−^ Drosophila* (Figure 3A). Consistent with these findings, while no significant differences were observed in the specific activities of complexes I and IV in 7-day old *dOpa1^+/−^* and *dOpa1^+/+^* flies, complexes II (38% reduction, p = 0.001) and III (37% reduction, p = 0.026) activities were significantly attenuated in *dOpa1^+/−^ Drosophila* (Figure 3B). In 35 d.o. *dOpa1^+/−^* flies, a similar impairment of enzymatic activities in complexes II (37% decrease, p = 1.97e-07) and III (28% decrease, p = 0.008) was observed. In addition, moderate (10%), but significant (p = 0.02) decline of complex IV activity was observed in 35 d.o. *dOpa1^+/−^* flies (Figure 3C). Our result also showed that there was a gender difference in the complex II activity. Heterozygous mutation of *dOpa*1 in males caused more significant inhibition of the complex II activity at 5 week of age (Supplemental Figure S1).

Mitochondria are not only the major sources of ROS production, but the complexes are also vulnerable targets of ROS [19]. To investigate whether the decreased complex activities cause increased ROS production or increased ROS inhibits the complex activities in the *dOpa1^+/−^* mutant files, we studied their mitochondrial aconitase activity. In the 7-day old flies, no significant differences in mitochondrial aconitase activities were observed between *dOpa1^+/−^* and *dOpa1^+/+^* flies (data not shown). However, in the 35-day old *dOpa1^+/−^* flies, mitochondrial aconitase activities were reduced by 22% relative to age-matched controls (Figure 3D). Furthermore, after reactivation with dithiothreitol and iron, aconitase activity was restored to an even higher level in *dOpa1^+/−^* flies. This result suggests that ETC dysfunction is the primary cause. Since the inhibition of aconitase activity is only observed in the older flies and the inhibition was reversible, increased ROS may enforce a vicious cycle and further lead to mitochondrial dysfunction.

### The shortened lifespan of heterozygous *dOpa1* mutants is extendable through the administration of antioxidants in male flies, but not in female flies

**Figure 4:**
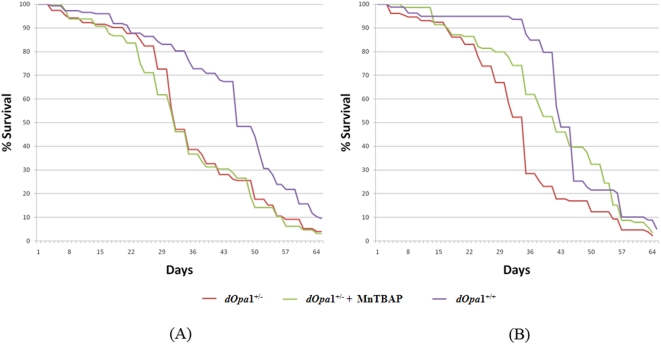
The shortened lifespan of heterozygous *dOpa1* mutants is extendable through the administration of antioxidants in male flies, but not in female flies. *dOpa1^+/−^* mutants and *dOpa1^+/+^* controls were transferred to fresh food every two to three days while aging. Male and female *dOpa1^+/−^ Drosophila* and *dOpa1^+/+^Drosophila* were separated and maintained on with and without 100 µM MnTBAP antioxidant food. Longevity assays were performed. MnTBAP feeding significantly extended lifespan of male *dOpa1^+/−^ Drosophila* (A), but not in the females (B).

Exogenously supplemented antioxidant can detoxify ROS and may retard aging. We have shown that antioxidant treatments were able to ameliorate ROS damage-induced glossy eye phenotype in *dOpa*1 mutant large clones [14]. To study if antioxidant administration can reverse the observed reduced lifespan of the *dOpa1^+/−^* mutant strain, we added MnTBAP to the fly food and generated the survival curves for *dOpa1^+/−^* flies. Since males and females of the same species may differ in their responses to interventions that affect lifespan [20], we separated male and female flies in the survival study. Indeed we observed sex-specific effects of antioxidant on lifespan in *dOpa1^+/−^* flies. While MnTBAP treatment showed no consequences on lifespan of female *dOpa1^+/−^* flies, significantly increased lifespan (50% survival time increased by 7 days) was observed in antioxidant-fed male *dOpa1^+/−^* flies (Figure 4).

### Heterozygous mutation of *dOpa1* results in highly irregular and dysmorphic mitochondria in the skeletal muscle of adult *Drosophila*

**Figure 5:**
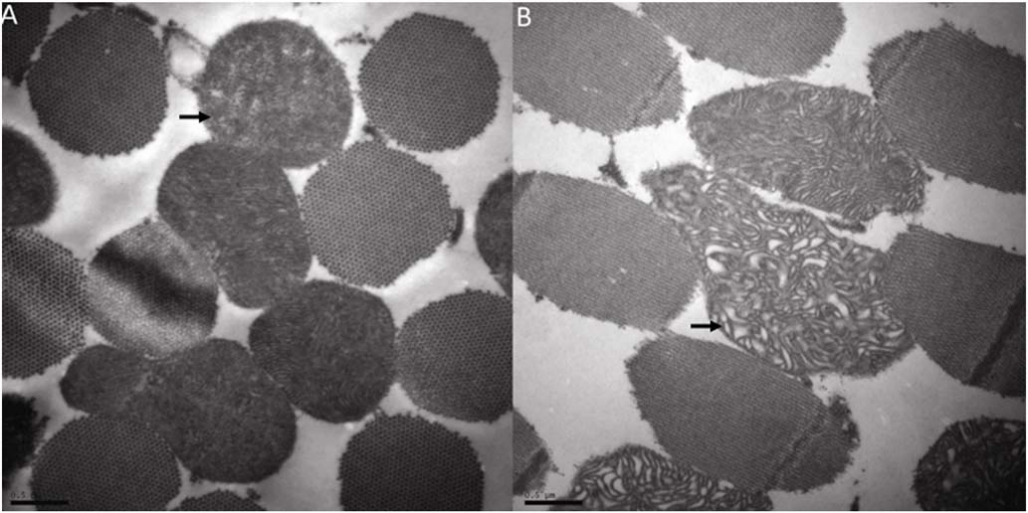
*dOpa1* ^+/−^ mitochondria are highly irregular and dysmorphic in the muscle. Tissues from adult *Drosophila* were analyzed by transmission electron microscopy. Mitochondrial phenotypes in muscle (A, B). The *dOpa1* ^+/−^ mitochondria were highly irregular and dysmorphic (Left) compared to wild type mitochondria (Right). Bar = 0.5 µm.

We previously described morphologically perturbed mitochondria in *dOpa1^+/−^* cells of ‘large clone’ *Drosophila* compound eyes [14]. Since OPA1 is ubiquitously expressed, it is interesting to investigate if *dOpa1* mutation also leads to morphological aberrancy of mitochondria in other organs than the eyes. We used TEM analysis (Figure 5) and found that the *dOpa1* ^+/−^ mitochondria were highly irregular and dysmorphic (Left) compared to *dOpa1* ^+/+^ mitochondria (Right) in the muscle. This result suggests that heterozygous mutation of *dOpa1* also affects the mitochondrial dynamics in *Drosophila*.

## Discussion

OPA1 is involved in mitochondrial fusion, cristae organization, control of apoptosis, ROS production and mutations in *OPA1* genes lead to DOA. Using the *Drosophila* model, we demonstrated that heterozygous mutation of *dOpa1* resulted in a shortened lifespan, enhanced production of ROS and boosted sensitivity to oxidative stress. Impairment of respiratory chain complex activities and age-dependent defects in aconitase activity were also observed, which is associated with irregular and dysmorphic mitochondria in the skeletal muscle. In male *dOpa1^+/−^* flies, shortened lifespan can be increased by administration of antioxidants.

Mitochondrial energy metabolism and ROS toxicity are important factors affecting *Drosophila* aging. The *Indy* mutant restricting the availability of calories lived longer [21]. Transgenic flies expressing increased Cu/ZnSOD and catalase [22] displayed extended lifespan. OPA1 is an important regulator of mitochondrial fusion and provide protection for the mitochondria from damage. OPA1 mutations in DOA patients have been associated with mitochondrial fragmentation [23], [24], mtDNA copy number reduction [25] and deletions [12], reduced ATP generation capability [26], [27], and increased susceptibility to apoptosis [28]. All these suggest OPA1 insufficiency relates to mitochondrial dysfunction, a well-known factor contributing to accelerated senescence, implicating the involvement of OPA1 in aging. Indeed, in our *Drosophila* model, lifespan of the *dOpa1^+/−^* mutants was significantly reduced, demonstrating the important role of OPA1 in longevity. We also observed increased mitochondrial ROS levels and production rates in *dOpa1^+/−^* adult *Drosophila*. Excessive mitochondrial ROS can shorten the lifespan by directly impairing mitochondrial genome, lipids of the inner membrane, mitochondrial protein activity and/or affecting nuclear gene transcription.

In our *dOpa1^+/−^ Drosophila* model, the most severely damaged electron transport chain unit is complex II, suggesting that complex II is an important determinant of *Drosophila* lifespan. This result is consistent with a comprehensive study of the lifespan in *Drosophila* with RNAi-inactivation of nuclear genome-encoded mitochondrial complexes I–V genes. The study shows one third of the RNAi lines that exhibited altered lifespan resulted from inactivation of complex II-encoding genes (David Walker, UCLA, personal communication).

In Hela cells, inhibition of Drp1 modifies the pattern of OPA1 isoforms, results in dysfunction of OPA1 and leads to a significant decrease in complex II respiration with complex I function intact [29]. The correlation between OPA1 defect and complex II dysfunction in both flies and Hela cells suggests that the mitochondria structure is important for integrity of complex II functions.

Mitochondrial complex II oxidizes succinate to fumarate and passes the electrons into the quinine, thus serving as the only mitochondrial membrane complex involved in both electron transport and the tricarboxylic acid cycle. It was shown that complex II defect alone is associated with an increased level of ROS [30]. Mutation in *sdhB* in *Drosophila*, encoding for subunit B of complex II, displays structural abnormalities in the mitochondria, causes an increased level of mitochondrial hydrogen peroxide, and is associated with hypersensitive to oxygen and shortened lifespan [30]. In *Caenorhabditis elegans*, a mutation in mev-1, encoding for a subunit of complex II, also resulted in augmented ROS production and shortened lifespan [31], [32]. In fact, the short-lived mutant exhibited increased level of nuclear damage, demonstrating mitochondrial derived oxidants may also be a significant source of overall genomic instability [33]. Whether OPA1 deficiency leads to nuclear complex II gene transcriptional reduction, damage to complex assembly or protein function remains to be elucidated.

In contrast to our data, in OPA-RNAi MEF cells, knock down of OPA1 induced a severe reduction in respiration, and oxygen consumption for complexes I, III and IV were all compromised [34]. Fibroblast cells derived from DOA patients with missense mutation showed a significant impairment of oxidative phosphorylation (OXPHOS), mostly mediated by complex I [27]. Furthermore, Co-immunoprecipitation experiments revealed a direct interaction between OPA1 and complexes I, II, and III, but not IV [27]. The discrepancy might be associated with the type of cells investigated — cultured fibroblast cells preferentially use anaerobic glycolysis for energy production and so mitochondrial OXPHOS in MEF might be metabolically different from that *in vivo*. In addition, different *OPA1* mutations can also result in variable energy defects [35], [36]. In our experiments, mutant *dOpa*1 *Drosophila* were generated by P-element or transposon insertion and resulted in *dOpa*1 haploinsufficiency.

ROS can damage the electron transport chain. The respiratory chain malfunction can also lead to increased ROS production, constituting a reinforcing vicious cycle and possibly resulting in catastrophic breakdown of mitochondrial function [37]. In the *dOpa1^+/−^* flies, both increased ROS levels and compromised ETC functions were observed. It is intriguing to investigate which serves as the primary cause. To distinguish between these two possibilities, we studied mitochondrial aconitase activity. At one week of age, heterozygous mutation of *dOpa*1 caused a significant decrease in the complex activities, but aconitase activity was normal, suggesting that dysfunction of ETC is the primary cause. However, we also observed an age-dependent aconitase activity decline and an elevated aconitase protein level in *dOpa1^+/−^* flies after reactivation at 5 weeks of age, suggesting that the aconitase activity reduction was the consequence of inactivation of existing enzyme by increased ROS. The elevated amount of extant aconitase in *dOpa1^+/−^* flies might reflect the physiological responses to synthesize more aconitase protein to compensate for the reduction in enzymatic activities. These data indicate that decreased complex activities are the primary underlying mechanism and the secondary increase of ROS reinforces vicious cycle and results in catastrophic mitochondrial dysfunction, including decrease in ATP production. The gradual accumulation of ROS and resultant chronic damage to the mitochondria is consistent with the progressive manifestation of phenotypes in OPA1-related DOA patients.

Gender is an important and profound factor in aging and lifespan and it is predicted that the longer-lived gender should have lower levels of oxidative stress. In both mammals and flies, females generally have a longer lifespan, possibly due to the sub-optimal mitochondrial function in the males [40]. The insulin/IGF pathway [19], [41] and JNK-dependent signaling [42] are important regulators for tolerance to oxidative stress and longevity in *Drosophila* and both cascades can be activated by ROS. Generally, females respond better to antioxidant interventions. The lifespan extension impacts of human SOD over-expression in *Drosophila* were more obvious in the females in six out of ten different genetic backgrounds studied while only one male background showed significant increase [43]. Interestingly, the antioxidant rescue of lifespan for heterozygous *dOpa*1 mutants is only effective in the males, but not in the females in our study, which is consistent with the complex II activity. The gender-specific variation of antioxidant influence can also be explained by differences in feeding habits and/or distinct responses to intracellular redox status changes between male and female flies.

In summary, OPA1 deficiency causes malfunction of ETC and results in elevated production of ROS, which in turn can further impaired the complex activity and mitochondrial bioenergetic capability and consequently accelerate aging in *dOpa*1^+/−^ mutants. Whether OPA1 insufficiency causes ETC dysfunction and induces augmented ROS generation is independent of its role in mitochondrial dynamics and apoptosis, as suggested in the *C. elegans* DOA model [44], requires furthering elucidation. In the future, the observations from the *Drosophila* DOA model can be extended to human DOA subjects and have the potential to establish a new model for longevity. Data presented in this study also suggest that antioxidants can sequester ROS and may hold potential as an effective therapeutic agent in this condition.

## Materials and Methods

### *Drosophila* stocks

y[d2] w[1118] P{ry[+t7.2] = *ey*-FLP.N}2; P{ry[+t7.2] = neoFRT}42D PBac{WH}CG8479^f02779^ (*dOpa1^+/−^*) and y[d2] w[1118] P{ry[+t7.2] = *ey*-FLP.N}2; P{ry[+t7.2] = neoFRT}42D PBac{WH}CG8479^f03594^ (*dOpa1^+/+^*) *Drosophila* were used in this study. These stocks were established in a previous study [14]. *dOpa1^+/−^* mutant type and *dOpa1^+/+^* control were transferred to new food every two to three days while aging. *dOpa1^+/−^* ^(antiox)^ *Drosophila* and *dOpa1^+/+^* ^(antiox)^ *Drosophila* are heterozygous mutant or wild-type *Drosophila* and were kept on antioxidant food.

### Longevity assay and *Drosophila* husbandry

Lifespan was determined under standard conditions for *Drosophila* husbandry (25°C, 50% Humidity, 12 hour light cycle) as described before [46]. *Drosophila* were collected over a 24 hour period and aged in vials containing standard *Drosophila* media (40–50 *Drosophila*/vial; same sex) and transferred every 3–6 days to fresh *Drosophila* media.

### ROS measurements

Adult *Drosophila* were collected within 12 h of emergence under light CO2 anesthesia, and transferred into vials with food in groups of 15, sexes separate. *Drosophila* were tested for the rate of superoxide anion production as well as levels of superoxide using MitoSOX (Invitrogen) based on previously established methods [14]. Briefly, tissue homogenates of 40 adult *Drosophila* were stained with MitoSOX (2.5 mM), and fluorescence was performed at 510 nm exciation/580 nm emission via a Perkin Elmer L20B luminescence spectrometer.

### Paraquat survival

Adult *Drosophila* were collected within 12 h of emergence under light CO_2_ anesthesia, and transferred into vials with food in groups of 15 *Drosophila* per vial, sexes separate. Seven days after emergence, *Drosophila* were tested for resistance to paraquat (Sigma), based on previously established methods [46]. *Drosophila* were starved for 2 h by placing them in empty vials, and then transferred into test vials containing filter paper disks soaked with 20 mM paraquat and 5% sucrose or 5% sucrose alone as a control. The starvation treatment ensures that initial ingestion of paraquat does not vary between lines because of differences in feeding status. Numbers of living *Drosophila* were recorded at 24 h intervals.

### Mitochondria isolation and respiration

Mitochondria were isolated as described before [46]. Respiration rates were determined by oxygen consumption using a Clark-type electrode and metabolic chamber containing 650 µL of reaction buffer consisting of 225 mM mannitol, 75 mM sucrose, 10 mM KCl, 10 mM Tris-HCl and 5 mM KH_2_PO_4_ (pH 7.2) at 25°C. Mitochondrial ATP production rates were calculated from ADP consumption rates during state III respiration.

### Mitochondrial enzyme assays

Citrate synthase activity was analyzed by the reduction of 5,5′-dithiobis-2-nitrobenzoic acid at 412 nm in the presence of acetyl-CoA and oxaloacetate [47]. Aconitase activity was measured by monitoring conversion of citrate into α-ketoglutarate at 340 nm at 25°C using the coupled reduction of NADP to NADPH [48]. Aconitase was reactivated by incubation with 2 mM dithiothreitol and 0.2 mM ferrous ammonium sulfate before repeating the enzymatic activity assays.

### Mitochondrial respiratory chain complex I–IV activity assay

Complex I (NADH dehydrogenase) activity was determined as the rotenone-sensitive NADH oxidation at 340 nm, using the coenzyme Q analogue 2, 3-dimethyl-5-methyl 6-n-decyl-1, 4-benzomethyluinone (DB) as an electron acceptor [47], [49]. The activity of complex II (succinate dehydrogenase) was analyzed by tracking the secondary reduction of 2,6 –dichlorophenolindophenol by ubiquinone-2 at 600 nm [47], [49]. Complex III (cytochrome *bc_1_* complex) activity was determined by measuring the reduction of cytochrome c at 550 nm with reduced decylubiquinone [47], [49]. Complex IV (cytochrome c oxidase) activity was measured by monitoring the oxidation of reduced cytochrome c as a decrease of absorbance at 550 nm [47], [49]. Complex I–IV activities were normalized by citrate synthase activity and then used in the analysis.

### Transmission electron microscopy (TEM)

TEM was performed as previously described [14]. Briefly, adult *Drosophila* were fixed overnight in 4% paraformaldehyde at 4°C and transferred to a post-fixation solution of 1% glutaraldehyde for 1 hour, then rinsed in PBS and placed in 1% osmium tetroxide for 20–60 min. The samples were dehydrated by ethanol and propylene oxide immersion. A flat-embedding procedure was used followed by trimming of the tissue block with a single-edged razor blade under a dissecting microscope (Nikon). A short series of ultrathin (60–80 nm) sections of the whole flies was cut from each block with an ultramicrotome (Reichert-Jung) and serial sections were collected on mesh and formvar-coated slot grids. The sections were stained with uranylacetate and lead citrate to enhance contrast. Skeletal muscle was examined with a Philips CM-10 transmission electron microscope and images of ommatidial units were captured with a Gatan digital camera.

## Supporting Information

Figure S1 Heterozygous dOpa1 mutation causes an age-dependent gender-specific difference of complex II activity decline.
(0.08 MB JPG)
