# Supplementary figures and images for: Heterozygous Mutation of Opa1 in Drosophila Shortens Lifespan Mediated through Increased Reactive Oxygen Species Production
Source: PLoS One. 2009 Feb 16;4(2):e4492. doi: 10.1371/journal.pone.0004492 (PMC2637430; doi:10.1371/journal.pone.0004492)

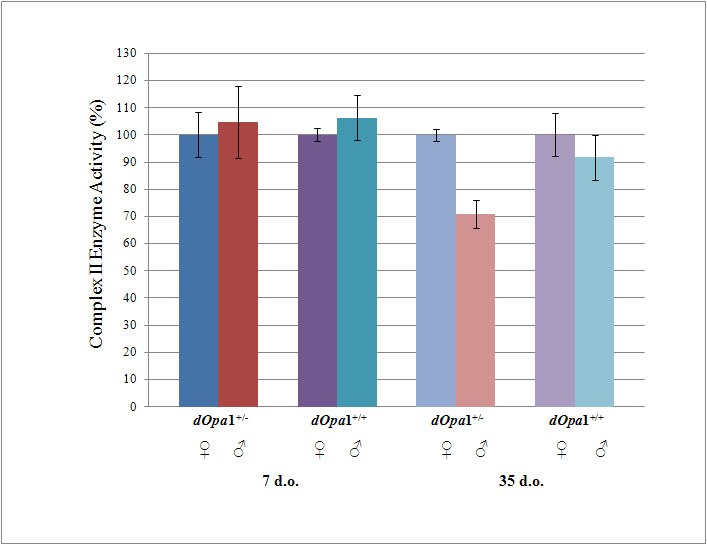

Supplement: Figure S1 — Heterozygous dOpa1 mutation causes an age-dependent gender-specific difference of complex II activity decline. (0.08 MB JPG) [file pone.0004492.s001.jpg]
